# Relationship between mitochondrial changes and seed aging as a limitation of viability for the storage of beech seed (*Fagus sylvatica* L.)

**DOI:** 10.7717/peerj.10569

**Published:** 2021-01-19

**Authors:** Arleta Małecka, Liliana Ciszewska, Aleksandra Staszak, Ewelina Ratajczak

**Affiliations:** 1 Laboratory of Biotechnology, Institute of Molecular Biology and Biotechnology, Adam Mickiewicz University, Poznan, Poland; 2 Department of Molecular and Cellular Biology, Institute of Molecular Biology and Biotechnology, Adam Mickiewicz University, Poznan, Poland; 3 Laboratory of Plant Physiology, Department of Plant Biology and Ecology, Faculty of Biology, University of Bialystok, Bialystok, Poland; 4 Institute of Dendrology, Polish Academy of Sciences, Kórnik, Poland

**Keywords:** Mitochondria, Seed storage, Common beech, Antioxidants

## Abstract

Aging is one of the most fundamental biological processes occurring in all forms of eukaryotic life. Beech trees (*Fagus sylvatica* L.) produce seeds in intervals of 5–10 years. Its yearly seed yield is usually very low, so there is a need for long-term seed storage to enable propagation of this species upon demand. Seeds for sowing must be of high quality but they are not easy to store without viability loss. Understanding the mechanism responsible for seed aging is therefore very important. We observed the generation of reactive oxygen species (ROS) in mitochondria of embryonic axes and cotyledons of beech seeds during natural aging. The presence of ROS led to changes in compromised mitochondrial membrane integrity and in mitochondrial metabolism and morphology. In this study, we pointed to the involvement of mitochondria in the natural aging process of beech seeds, but the molecular mechanisms underlying this involvement are still unknown.

## Introduction

During storage, seeds lose viability due to aging processes. Seed aging processes are controlled by temperature, the oxygen level and moisture conditions, at which seeds are stored but may also be associated with various metabolic and biophysical conditions (Bailly, 2004; Walters, Hill & Wheeler, 2005; Walters, Ballesteros & Vertucci, 2010; Ballesteros & Walters, 2011; Bewley et al., 2013). The main theory of aging is the ‘free radical theory’ proposed by Harman (2006). It postulates that damage accumulation caused by free radicals such as: lipid peroxidation, degradation of RNA and DNA, and inhibition of protein synthesis is the underlying mechanism of aging in all living organisms (Kibinza et al., 2011; Bellani et al., 2012; Chen et al., 2013; Xia et al., 2015). Additionally, changes in intercellular ROS levels, energy production or redox status by mitochondrial function or dysfunction triggers various responses that regulate mitochondrial and nuclear gene expression pivotal to seed aging (Yin et al., 2016). Seed aging is linked to a decrease in the metabolic rate and ends in seed death due to accumulated oxidative degradation of cellular constituents (Bailly, 2004; Kranner et al., 2006; Kibinza et al., 2011; Xin et al., 2014; Ratajczak et al., 2015). Because beech seeds are produced in large amounts irregularly every 5–10 years and climate changes also influence this cycle, the proper storage of beech seeds has become an important issue not only for gene banks but also for forestry industries. Different models for climate change indicate that beech is one of the most important species that may cover new areas in response to deep climate change in Central Europe (Dyderski et al., 2018).

**Figure 1:**
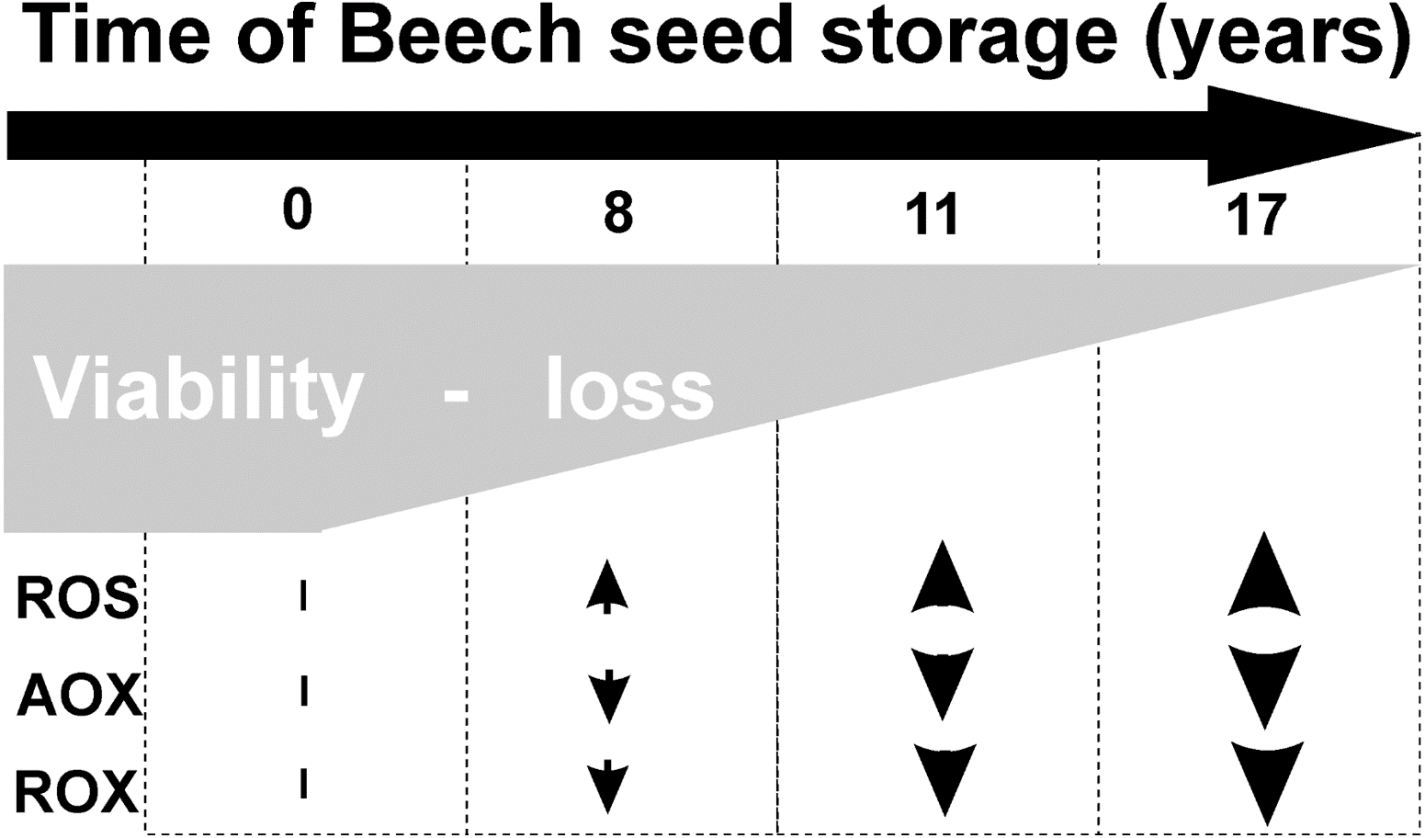
Characteristic of changes in beech seeds during long-term storage.

Common beech seeds belong to the intermediate category of seeds (Pukacka & Wójkiewicz, 2003). In earliest studies, we have shown that losses of germination capacity and viability of beech (*Fagus sylvatica* L.) seeds during storage (8–17 years) are associated with an increasing: ROS levels and oxidative stress conditions in seed cells (Fig. 1). Therefore, we observed changes in beech seed cells characteristic of oxidative stress, such as increased levels of superoxide radicals (O_2_^−•^), hydrogen peroxide (H_2_O_2_) and hydroxyl radicals (OH•) (Ratajczak et al., 2015) limited activity of the antioxidant system (Pukacka & Ratajczak, 2007; Pukacka & Ratajczak, 2014) (Fig. 1), changes in protein metabolism (Ratajczak et al., 2015), decreased levels of oligosaccharides from the sucrose and raffinose family (RFO) (raffinose and stachyose) and an increase in *α*-galactosidase activity (Pukacka, Ratajczak & Kalemba, 2009); and redox changes in cells (Pukacka & Ratajczak, 2007; Ratajczak, Dietz & Kalemba, 2018). We could not clearly identify the main cause of aging of beech seeds during their long-term storage or locate the cellular compartment that plays a major role in such important processes. Therefore, our next goal was to perform research at the subcellular level to identify the place from which the seed aging process begins. In our publication, we focus on determining whether mitochondria are involved in the natural seed aging process in order to be able to offer a better way to store beech seed stocks in the future.

Mitochondria, due to the presence of the respiratory chain, are the main sites for ROS generation in the cell (Møller, 2001; Howell, Millar & Whelan, 2006; Navrot et al., 2007) and play an important role in cell signaling. We think, as do some other authors (Daum et al., 2013; Dunn et al., 2015; Ratajczak et al., 2019), that the process of seed aging is induced by ROS and is associated with mitochondria. Mitochondria are closely involved in aging through changes occurring within these organelles, but the underlying molecular mechanisms are largely unknown. The most mobile ROS is H_2_O_2_, which may thus interact with various components of the cell (Ratajczak et al., 2015). The major phospholipid components of the mitochondrial membrane are rich in unsaturated fatty acids that are potentially susceptible to oxygen radical attack. Malondialdehyde (MDA) is the principal product of polyunsaturated fatty acid peroxidation (Slimen et al., 2014) and is considered an important marker of oxidative damage to cell membranes (Fu, Ahmed & Diederichsen, 2015). To maintain steady-state concentrations, ROS mitochondria have their own antioxidant system composed of enzymatic and nonenzymatic antioxidants (Xia et al., 2015). Catalase (CAT) is a key enzyme for seed protection against ROS-induced aging (Kibinza et al., 2011; Ratajczak et al., 2015). We demonstrated that storage time-dependent decreases in CAT activity occur in the embryonic axes and cotyledons of beech seeds. The decrease in CAT activity was strongly negatively correlated with the germination capacity of beech seeds during storage (Ratajczak et al., 2015). As a result of storage, the activity of the antioxidant system in seed cells is reduced (Xia et al., 2015; Mao et al., 2018). Decreased activity of the antioxidant system increases ROS production and reflects damage to the structures of mitochondria (Xia et al., 2015; Li et al., 2017), especially mitochondrial membranes. We wanted to determine whether mitochondria can be the main initiators of beech seed aging. For this purpose, in our research, we visualized changes in the structure of mitochondria, antioxidant enzyme activity of CAT, H_2_O_2_ level and degree of lipid peroxidation in beech seeds during natural aging. We identified these changes related to seed aging at the subcellular level in material stored for 8–17 years.

## Materials & Methods

Seeds of *Fagus sylvatica* L. were collected from a single tree growing in the Kornik Arboretum (Poland) from the cropping years 2000 (seed stored for 17 years), 2006 (seed stored for 11 years), 2009 (seed stored for 8 years) and 2017 (control). The experiments were performed in 2017; therefore, the seeds of this year are regarded as controls . Each seeds were desiccated to 7–8% of the water content, seeds were stored at −10 °C in tightly sealed containers.

A germination test was performed according to method described earlier by Ratajczak et al. (2015).

### Isolation of mitochondria

Mitochondria from beech seeds (from 20 embryonic axes and 5 cotyledons) were isolated using methods described by Malecka et al. (2009) in Perccol at 40,000×g for 30 min. Afterwards, the mitochondrial fractions were carefully collected and washed to remove Percoll in a 20-fold volume of the buffer K_2_HPO_4_/KH_2_PO_4_ (Potassium phosphate buffer) with 0.35 M sucrose, 20 mM MOPS (4-Morpholino propanesulfonic acid), pH 7.2. The purified mitochondria were resuspended in the same buffer with 0.35 M sucrose. For further analysis mitochondria extracted from embryonic axes and cotyledon seeds were used.

### Hydrogen peroxide determination

Hydrogen peroxide was determined according to Patterson, Macrae & Ferguson (1984). The decrease in absorbance was measured at 508 nm. Hydrogen peroxide was calculated by the standard curve for a 0.5–25 µM concentration range.

### Determination of CAT activity

The activity of CAT in the mitochondrion was determined by directly measuring the decomposition of H_2_O_2_ at 240 nm as described by Aebi (1983).

### Measurement of lipid peroxidation

Malondialdehyde (MDA) content was determined by reaction with thiobarbituric acid (TBA) as described by Heath & Packer (1968).

### Ultrastructural observation of mitochondria

Mitochondria from the embryonic axes and cotyledons of beech were submerged for 20 min in 0.05 M KH_2_PO_4_/K_2_HPO_4_ buffer (pH 7.5) containing 0.35 M saccharose and 2.5 µM Rhodamine 123 in the dark for 30 min (Johnson, Walsh & Chen, 1980, modified). After two rinses with 50 mM phosphate buffer (pH 7.5) with 0.35 mM saccharose, samples were centrifuged at 4,000 rpm at a temperature of 4 °C. The mitochondria were suspended in the above-mentioned buffer and viewed using a confocal microscope (the model Zeiss LSM 510, Axioverd 200 M, Jena, Germany) equipped with filter set no. 10 at an excitation of 488 nm and emission of 500–550 nm.

### Protein quantification

Total soluble protein contents were determined according to the method of Bradford (1976).

### Statistical analyses

The experiments were made in triplicate, differences were considered to be statistically significant if *p* < 0.5 for statistical analysis STATISTICA (StatSoft Poland, Kraków, Poland) software were used. Statistical analyses were carried out using ANOVA and the Tukey-Kramer HSD test.

## Results

### Seed germinability

**Figure 2:**
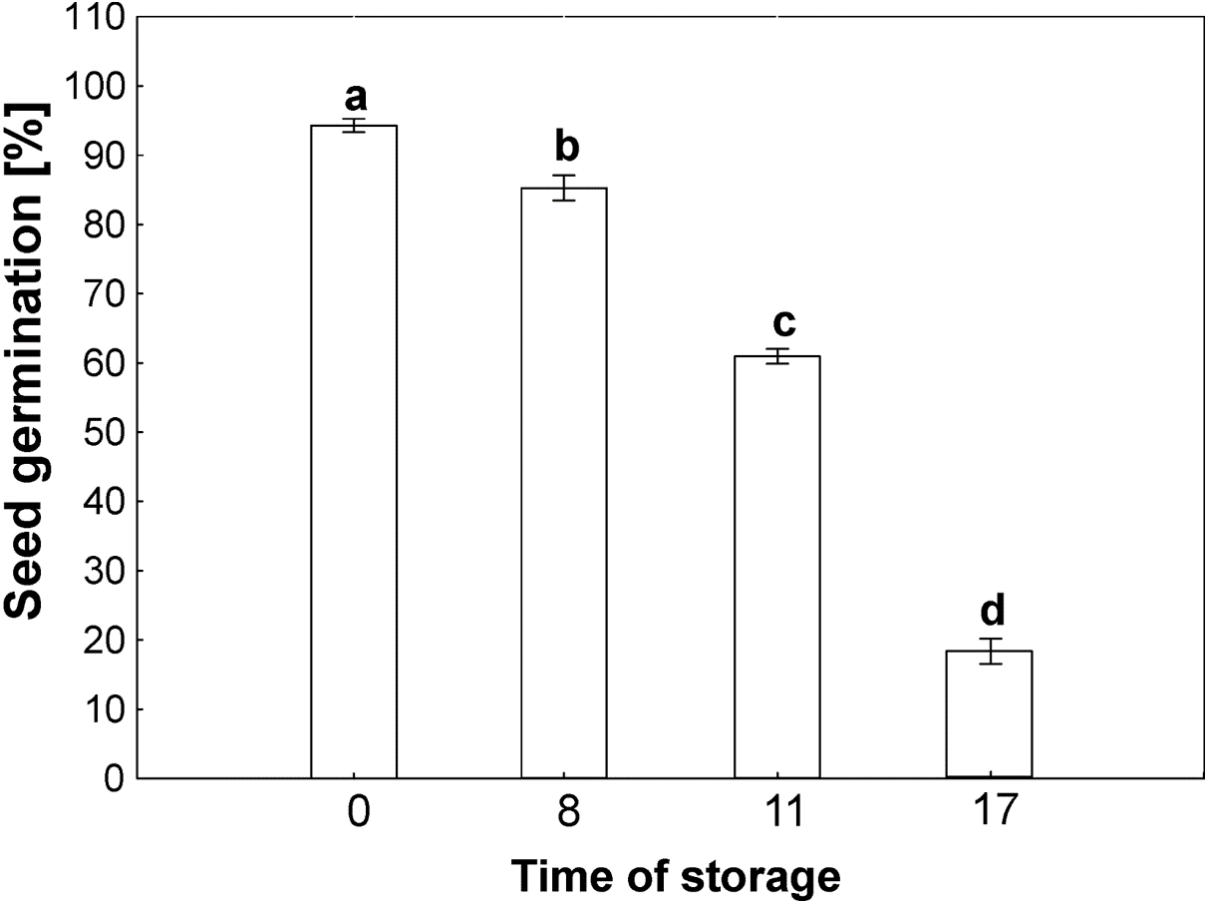
Seed germination of common beech (*Fagus sylvatica* L.) after storage for 0, 8, 11 and 17 years under optimal conditions. Statistically significant differences are indicated with different letters when *p* ≤ 0.05. The data are means ± SD of four biological replicates.

The germinability of common beech seeds after harvest reach 100% in year of harvest. Due to the duration of storage, the beech seeds showed different levels of germinability, as shown in Fig. 2. The germinability of control seeds stored for 2 years reached 100%. Seventeen years of storage was almost lethal for beech seed germination, which was 10% after this period of time. Seeds stored for 8 years reached 90% germination. Storage for 11 years caused a decrease in germinability to 65%.

### Determination of H_2_**O**_2_ level

**Figure 3:**
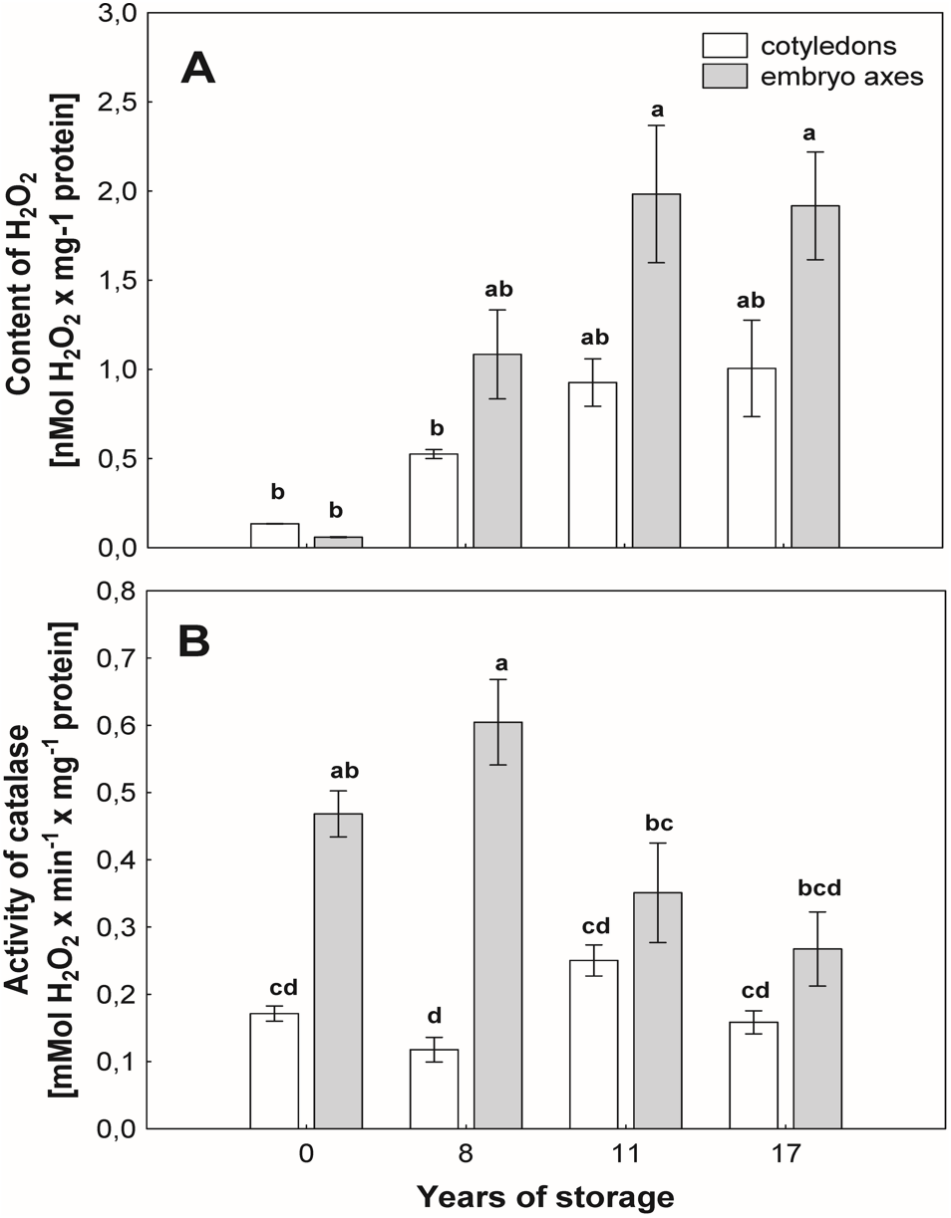
The levels of hydrogen peroxide (*H*_2_*O*_2_) in mitochondria from the embryonic axes and cotyledons common beech (*Fagus sylvatica* L.) seeds stored for 8, 11 and 17 years under optimal conditions (A). Catalase (CAT) activity measured in mitochondria from the cotyledons and embryonic axes of common beech (*Fagus sylvatica* L.) seeds stored for 8, 11 and 17 years under optimal conditions (B). Statistically significant differences are indicated with different letters when *p* ≤ 0.05. The data are the means ± SD of four biological replicates.

With the aging of seeds, we observed an increase in the level of H_2_O_2_ in mitochondria from the cotyledons and axes, especially in the beech seeds stored for longer durations (Fig. 3A). In mitochondria from the cotyledons of seeds stored for 17 years, 2-fold and greater-than-7-fold increases in the level of H_2_O_2_ were observed in relation to the mitochondria from the cotyledons of seeds stored for 8 years and control seeds, respectively (Fig. 3A). Similarly, mitochondria from the embryos of seeds stored for 17 years generated twice as much hydrogen peroxide as did those from seeds stored for 8 years and more than three times as much hydrogen peroxide than produced by control seeds (Fig. 3A).

### CAT assay

The activity of CAT (Fig. 3B), an enzyme that removes mitochondrial H_2_O_2_, was similar among mitochondria from cotyledons of beech seeds stored for all researched periods. However, some differences in the activity of this enzyme were observed in mitochondria isolated from the embryonic axes of beech seeds during storage. The maximum activity was recorded in mitochondria from the embryonic axes of beech seeds stored for 8 years, and this value was 3 times higher than that in the mitochondria embryonic axes of seeds stored for 17 years and approximately 20% higher than that in mitochondria from the youngest seeds, i.e., control seeds.

### Changes in mitochondrial membranes

**Figure 4:**
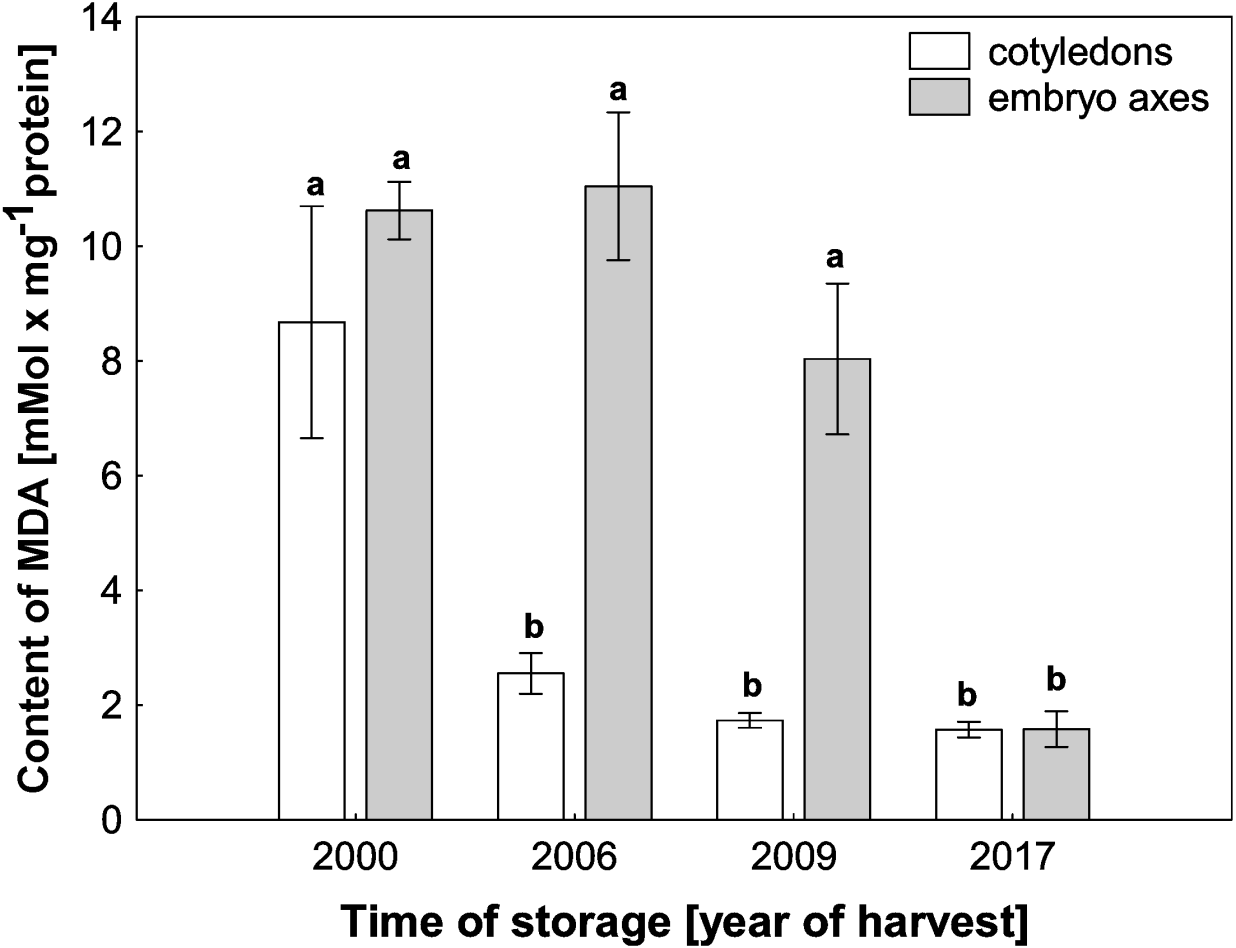
The levels of MDA in mitochondria from the embryonic axes and cotyledons of common beech (*Fagus sylvatica* L.) seeds stored for 8, 11 and 17 years under optimal conditions. Statistically significant differences are indicated with different letters. when *p* ≤ 0.05. The data are 430 the means ± SD of four biological replicates.

ROS cause oxidative stress conditions in cells and consequently contribute to the increase in membrane lipid peroxidation. In the mitochondria from cotyledons and embryonic axes of beech seeds stored for 17 years, we noticed a very high level of MDA, which was more than 5–6 times higher than that in mitochondria from the fresh beech seeds (control) (Fig. 4). At the same time, there was a gradual decrease in the degree of membrane lipid peroxidation in mitochondria from the cotyledons and embryonic axes of beech seeds stored for 11 and 8 years; this decrease was correlated with the H_2_O_2_ level.

### Structure of the mitochondrion in seeds during storage

**Figure 5:**
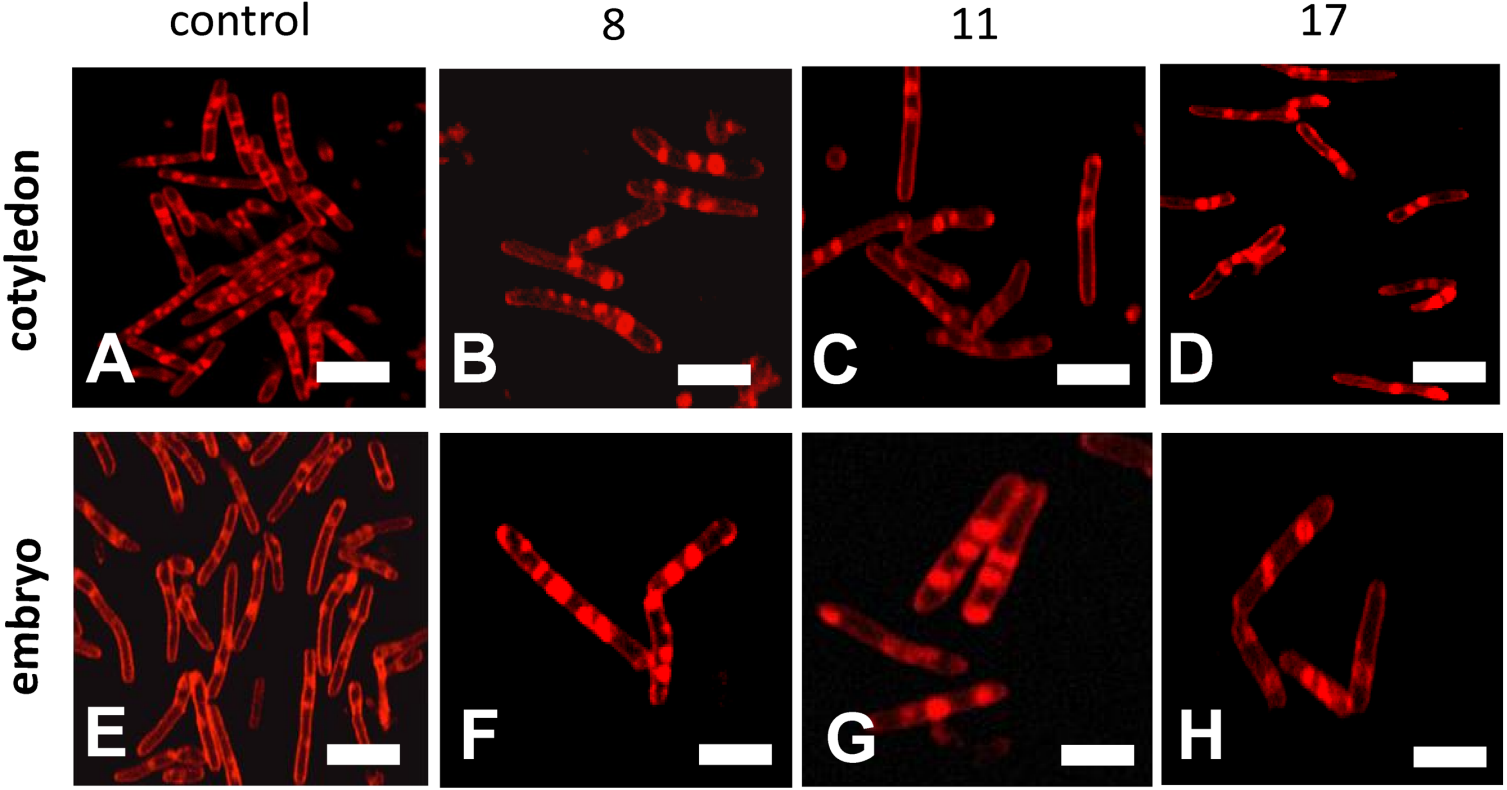
Fluorescent images of mitochondrial structural changes in the embryonic axes and cotyledons common beech (*Fagus sylvatica* L.) seeds stored for 8, 11 and 17 years under optimal conditions. Control seeds (A and E), seed stored for 8 (B and F), 11 (C and G) and 17 years (D and H).

Confocal microscopy observations of mitochondria showed that their ultrastructures changed progressively during the beech seed aging process (Fig. 5). The mitochondria isolated from the oldest cotyledons and axes of embryonic beech seeds stored for 17 years were smaller than those isolated from younger cotyledons and embryonic axes, i.e., those from seeds stored for 11 and 8 years, and had double membranes preserved but with a reduced amount of cristae. Mitochondria often occur singly or in small clusters of several organelles. In contrast, the mitochondria of control seeds were much larger than those of the oldest seeds and had an elongated shape and more numerous visible cristae. In addition, the mitochondria occurred in large aggregates.

## Discussion

During storage, seeds undergo aging processes, which decrease germination and viability (Cakmak et al., 2010; Kaewnaree et al., 2011; Moncaleano-Escandon et al., 2013). Our previous studies (Pukacka & Ratajczak, 2007; Ratajczak et al., 2015) and current tests confirm that beech seeds lose the ability to germinate as the length of their storage time increases (Fig. 1). Understanding the main cause and place of the initiation of the aging process is one of the most important areas of study, but little is known about how this process looks under the long-term storage of seeds of long-living organisms such as trees.

Mitochondria are cell structures in which ROS are produced under oxidative stress conditions and aging (Kowaltowski et al., 2009; Yin et al., 2016). In the normal metabolism of oxygen, ROS are produced as natural byproducts that play important roles in seed germination as message molecules (Li et al., 2017). Our research showed that in mitochondria from the cotyledons and embryonic axes of stored beech seeds, there was a several-fold increase in H_2_O_2_ levels during seed aging (Fig. 2A). Li et al. (2017) showed that the H_2_O_2_ levels in the cotyledons and hypocotyls of aging elm seeds increased on days 2 and 3 and decreased after 5 days, with a loss of seed vigor. Other authors (Xia et al., 2015) demonstrated increasing levels of H_2_O_2_ in imbibed oat seeds aged from 0 to 24 days and observed a decline in the mitochondrial H_2_O_2_ content after 32 and 40 days; this decline was most likely due to the destruction of the mitochondrial ultrastructure. Jimenez et al. (1998) showed that a decrease in mitochondrial integrity leads to H_2_O_2_ leakage from the mitochondria to the cytosol in pea plants during senescence. ROS levels should be tightly regulated by balancing production and scavenging, and the accumulation of ROS results in uncontrolled oxidative damage, such as lipid peroxidation. We observed that the increase in hydrogen peroxide in mitochondria from the cotyledons and axes of aging beech is accompanied by an increase in the level of MDA (Fig. 4), indicating the occurrence of oxidative damage. Similarly, Xia et al. (2015) observed that in imbibed (4% and 10%) oat seeds aged from 0 to 40 days; H_2_O_2_ generation increased MDA levels. The literature suggests that changes in mitochondrial structure are responsible for a decrease in the activity of antioxidant enzymes in old seeds (Mao et al., 2018). We observed in our research that the aging of beech seeds is accompanied by a decrease in CAT activity in mitochondria. Xin et al. (2014) indicated that the activities of mitochondrial antioxidant enzymes, such as SOD (superoxide dismutase), APX (ascorbate peroxidase), GR (glutathione reductase), MDHAR (monodehydroascorbate reductase) and DHAR (dehydroascorbate reductase), were significantly reduced in soybean seeds aged at 40 °C for 18 and 41 days. In imbibed oat seeds aged 8 days under high-moisture (16%) conditions, a decrease in the antioxidant potential was observed (Xia et al., 2015). The authors claim that the cause of this decrease may be the leakage of enzymatic and nonenzymatic antioxidants due to mitochondrial damage to the cytosol. Using transmission electron microscopy, Yin and coauthors (2016) showed that mitochondrial enzyme activity and oxygen consumption were paralleled by damage to the mitochondrial ultrastructure in aged seeds. The authors also noticed that in the mitochondria of these seeds, the ultrastructure was that of promitochondria, antioxidant enzyme activities were extremely low, and the respiratory capacity was limited. In our study, the observed changes in mitochondrial structure were associated with the presence of high levels of hydrogen peroxide and MDA. Cogliati, Enriquez & Scorrano (2018) reported that oxidative stress causes mitochondrial elongation, protecting mitochondria from degradation and promoting mitochondrial ATP (adenosine 5′-triphosphate) production. Xia et al. (2015) suggested that there are relationships between antioxidative systems and mitochondrial ultrastructure in aging seeds. They used transmission electron microscopy to observe that the mitochondrial ultrastructure of these seeds was damaged during 40 days of aging, and the degree of damage was related to the level of seed moisture (from 4 to 16%) until the cristae disappeared completely. Other authors have shown that as a result of endogenously produced ROS in the mitochondria of *Ulmus pumila L,* structural changes occur which are observed in the early stages of aging. Cogliati, Enriquez & Scorrano (2018) noticed that the morphology of mitochondria can change to match the needs of cells. When in aging organisms the amount of mitochondria that lose their ability to perform their functions increases so that the cellular repair mechanisms fail, programmed cell death takes place. In our research, we observed very large changes in the structure of the mitochondria with storage time, which were strongly related to the aging process.

## Conclusion

We believe that the aging processes are related to the mitochondria, and their careful analysis will allow us to understand them. We have shown that during the aging of beech seeds, the levels of hydrogen peroxide and MDA increase, while the activity of CAT decreases. At the same time, we observed changes in the structure of the mitochondria that indicated their decreased activity. The generation of ROS leads to changes in the structure of mitochondria and impairs their functioning, which triggers defense mechanisms that regulate both mitochondrial and nuclear expression of genes that are crucial in the aging process of seeds. Undoubtedly, the problem of aging mitochondria requires a wider range of research to understand the molecular basis of these changes.

##  Supplemental Information

10.7717/peerj.10569/supp-1 Supplemental Information 1 CAT, H2O2, MDA, and Germination data.

## Additional Information and Declarations

### Data Availability

The following information was supplied regarding data availability: The raw measurements are available in the Supplemental Files.
